# Narrative Review Concerning the Clinical Spectrum of Ophthalmological Impairments in Parkinson’s Disease

**DOI:** 10.3390/neurolint15010012

**Published:** 2023-01-26

**Authors:** Alina Zorina Stuparu, Sanda Jurja, Alexandru Floris Stuparu, Any Axelerad

**Affiliations:** 1 Department of Neurology, General Medicine Faculty, Ovidius University, 900470 Constanta, Romania; 2 Department of Neurology, St. Andrew County Clinical Emergency Hospital of Constanta, 900591 Constanta, Romania; 3 Department of Ophthalmology, General Medicine Faculty, Ovidius University, 900470 Constanta, Romania; 4 Department of Ophthalmology, St. Andrew County Clinical Emergency Hospital of Constanta, 900591 Constanta, Romania; 5 Department of Orthopedy and Traumatology, St. Andrew County Clinical Emergency Hospital of Constanta, 900591 Constanta, Romania

**Keywords:** Parkinson’s disease, ophthalmological impairments, optic, eye, vision

## Abstract

Ophthalmic non-motor impairments are common in Parkinson’s disease patients, from the onset of the neurodegenerative disease and even prior to the development of motor symptoms. This is a very crucial component of the potential for early detection of this disease, even in its earliest stages. Since the ophthalmological disease is extensive and impacts all extraocular and intraocular components of the optical analyzer, a competent assessment of it would be beneficial for the patients. Because the retina is an extension of the nervous system and has the same embryonic genesis as the central nervous system, it is helpful to investigate the retinal changes in Parkinson’s disease in order to hypothesize insights that may also be applicable to the brain. As a consequence, the detection of these symptoms and signs may improve the medical evaluation of PD and predict the illness’ prognosis. Another valuable aspect of this pathology is the fact that the ophthalmological damage contributes significantly to the decrease in the quality of life of patients with Parkinson’s disease. We provide an overview of the most significant ophthalmologic impairments associated with Parkinson’s disease. These results certainly constitute a large number of the prevalent visual impairments experienced by PD patients.

## 1. Introduction

The three primary symptoms of Parkinson’s disease are tremor at rest, stiffness, and bradykinesia [1]. The motor symptoms of early Parkinson’s disease are often asymmetric, and symptomatic remission with dopaminergic medications is substantial and long-lasting. Both familial parkinsonism and familial Parkinson’s disease may be inherited autosomal dominantly (with varied penetrance) or autosomal recessively.

Prior to the onset of motor symptoms, sleep disturbances are prevalent in early Parkinson’s disease. A common symptom is REM sleep behavior disorder. It is caused by the absence of normal muscular atonia throughout REM sleep and manifests as excessive movement while dreaming. Additional early nonmotor signs are constipation, and seborrheic dermatitis. Other early nonmotor signs involve constipation, seborrheic dermatitis, hyposmia, and dysautonomia, such as bladder failure.

Despite the fact that movement disorder is the most prominent symptom of PD, visual impairment is one of the non-motor symptoms reported in this condition. Numerous studies have shown that people with Parkinson’s disease exhibit a variety of visual abnormalities, such as color discrimination, visual acuity, contrast sensitivity, blurry vision, motion perception, and loss of vision [2]. This visual impairment is induced by the decrease of dopamine in the amacrine and inner plexiform cells of the retina in individuals with Parkinson’s disease [3]. Dopamine is a crucial biological substance required for brain control and retinal growth, visual signaling, and refraction function [4].

It has been shown that aberrant variations in dopamine and the loss of retinal amacrine cells contribute to abnormalities in the receptive characteristics of ganglion cells, resulting in defective informational perception in individuals with PD [5]. Ortuño-Lizarán demonstrated that Alpha-Syn was aggregated in the inner nuclear, inner plexiform, and ganglion cell layers of the intraretinal area [6]. Therefore, it may be argued that the buildup of phosphorylated Alpha-Syn is the only cause of the permanent loss of dopamine in the brain and retina of PD patients [7]. Furthermore, it was shown that iron plays a crucial role in the aggregation of Alpha-Syn and the reduction of dopamine neurons in the retinal cells of Parkinson’s disease patients [8]. These results show that the development of retinal biomarkers in PD would be a prerequisite for early detection of this neurodegenerative disorder.

The purpose of this article is to evaluate many of the least-recognized visual manifestations of Parkinson’s disease and to assess the research addressing these symptoms. We emphasize the clinical significance of these manifestations as initial disease indicators and potential outcome indicators, with a particular focus on visual processing impairments and oculomotor abnormalities.

In PD, visual manifestations are prevalent. According to a recent study, 78% of patients diagnosed with PD noted at least one difficulty involving their vision or visuospatial functioning, such as visual hallucinations, double vision, and freezing of gait (which appeared to be connected with deficient contrast sensitivity) [9,10]. Despite the fact that dry eyes and a decreased blink rate are the most prevalent ocular impairments in PD [11], numerous other difficulties with vision, visual processing, and ocular movement could also develop; these will be described in this review.

## 2. Materials and Methods

Our research used the academic electronic databases PubMed, Google Scholar, Web of Science, and Science Direct. Publications in English between 1970 and 2022 containing the terms “optic”, “ophthalmology”, and “ocular” in relation to “Parkinson’s disease” and “PD” were included in our selection. The selection of 75 articles was determined using data analysis. Manually added references from current research and case reports to the search. The titles of the documents were checked for accuracy, and duplicates were removed. 100 articles were detected in total; 15 were eliminated after the full-text screening, and 10 were eliminated because of duplication. Academic material on the ophthalmologic symptomatology of Parkinson’s disease fulfilled the eligibility conditions.

In order to confirm the inclusion of material important to Parkinson’s disease patients and their ophthalmological deficits, the authors manually verified the accuracy of the reference lists of the chosen literature. Papers were considered for inclusion if they met the following criteria: (1) they were published in English; and (2) they were original research publications, reviews, or case reports. Publications that fulfilled the following requirements were omitted: (1) citations and patents, (2) papers written in a language other than English, and (3) abstracts that lacked data.

## 3. Pathogenesis of Parkinson’s Disease

### 3.1. Pathogenesis

Parkinson’s disease is linked to the gradual death of dopaminergic neurons in the substantia nigra, resulting in hallmark motor aspects of the disease along with more widespread neuronal alterations that cause complicated and diverse nonmotor symptoms. Recent breakthroughs in PD genetics have highlighted the importance of mitochondrial dysfunction in PD development [12]. Impaired mitochondrial activity increases oxidative stress and might make cells more susceptible to this and other interconnected processes, such as excitotoxicity. Consequently, this pathway represents a potential treatment approach. Despite the fact that various therapies are helpful in treating the symptoms of PD, a deeper knowledge of the underlying pathophysiological pathways is essential to facilitate the development of innovative neuroprotective therapeutics.

### 3.2. Mitochondrial Dysfunction in Parkinson’s Disease

Mitochondria are plentiful in tissues with high metabolic requirements, such as the brain and muscle, because they play a critical role in energy metabolism. There is substantial evidence implicating mitochondrial damage, in the pathogenesis of Parkinson’s disease [13,14]. The substantia nigra is more susceptible to such deficiency, and extensive research reveals multiple variations in the expression of genes controlling energy consumption between dopamine neurons of the substantia nigra and elsewhere [15].

Mitochondria represents the key to a variety of processes believed to be fundamental to the pathogenesis of Parkinson’s disease. It is also believed that oxidative stress plays a crucial role in the etiology of Parkinson’s disease [16]. Oxidative stress might result from either increased exposure to free radicals or elevated risk and failure of the oxidative stress response. These two pathways could be impacted by mitochondrial malfunction. Firstly, the electron transport chain is a significant producer of free radicals-molecules with unpaired electrons, such as superoxide and hydroxyl radicals [16]. By removing electrons from surrounding molecules, they are extremely reactive and generate oxidative damage. Reduced electron transport chain activity increases the production of free radicals [17], to which mitochondrial proteins and DNA may be especially vulnerable. Second, poor mitochondrial activity increases oxidative stress susceptibility.

### 3.3. Oxidative Stress in Parkinson’s Disease

Oxidative stress is characterized as an imbalance involving reactive oxygen species generation and antioxidant protection [18]. Oxidative stress is a major cause of hereditary retinal dystrophies, but a lot of the molecular pathways that are involved are still not well understood [19,20,21]. Oxidative stress might induce abnormal cellular bioenergetics and initiate pathogenic processes that culminate in clinically detectable PD, as it was discovered to affect the retinal area [18]. Multiple hypotheses postulate that oxidative stress contributes to the pathogenesis of Parkinson’s disease, and that greater susceptibility to oxidative stress could make particular cell types more susceptible to damage in PD [22,23]. Protein oxidation, lipid peroxidation, and DNA damage may be caused by an excess of reactive oxygen and nitrogen species. This creates an unnecessary burden on the cellular mechanism necessary for the breakdown of damaged proteins, which is itself implicated in the etiology of Parkinson’s disease.

Multiple factors may contribute to an increase in oxidative damage in Parkinson’s disease: failure of the mitochondrial electron transport chain (ETC), defective antioxidant systems, increased exposure to environmental or endogenous causes of oxidative stress, and excitotoxicity. Multiple antioxidant systems, including glutathione, superoxide dismutase, and DJ-1, rigorously regulate the amounts of reactive oxygen species in normal dopaminergic neurons. Nonetheless, these procedures are frequently ineffective in Parkinson’s disease patients. As a result, researchers discovered significantly more mutations, particularly somatic deletions in mitochondrial DNA, in the substantia nigra of Parkinson’s disease patients compared to same-age controls, supporting the notion that nigral neurons are disproportionately more vulnerable to oxidative damage [24,25]. In Parkinson’s disease, the loss of protective factors against oxidative stress is frequent. DJ-1 is a protein complex with roles in transcriptional control and oxidative stress, and the decrease of its activity is considered to lead to the development of PD. DJ-1 defends from UV-related induction of apoptosis through the inhibition of the JNK1 upregulation, according to research [26,27]. NAD+ governs energy consumption, recovery of DNA damage, gene expression, antioxidant defense, and the response to stress. It has been discovered that neurodegenerative disorders and retinal degeneration are accompanied by a reduction in NAD+ levels [28,29]. The potential implications for health policy are enormous. The herbicide paraquat, for instance, induces oxidative stress [22,23], resulting in the death of dopamine neurons and motor impairments in rats [30]. Epidemiological studies have connected this chemical to an increased incidence of PD. In addition, it has been proposed that a diet heavy in animal fat may increase the incidence of Parkinson’s disease, potentially via altering redox equilibrium [31].

### 3.4. Parkinson’s Disease Evidence of Oxidative Injury

Brain autopsy investigations of biochemical markers for oxidative damage provide definitive proof of oxidative stress in Parkinson’s disease patients. Postmortem investigations indicate elevated amounts of malondialdehyde, cholesterol lipid hydroperoxides, and other indicators of lipid peroxidation and oxidative stress [32,33]. Enhanced immunostaining for 4-hydroxynonenal, a hallmark for membrane lipid peroxidation, indicates that the rise in oxidative damage to lipids in the substantia nigra happens in pigmented neurons [34]. Additionally, oxidative DNA degradation has been observed in the nervous system of PD patients in autopsies [34]. PD patients’ brain tissue exhibits increased numbers of protein oxidative damage based on a sparsely distributed increase in protein carbonyls [35]. Furthermore, elevated 3-nitrotyrosine and nitrated alpha-synuclein immunoreactivity have been found in Parkinson’s disease patients’ Lewy bodies [36]. Intracellular peroxynitrite generation causes Alpha--synuclein aggregation [37], and oxidative damage inhibits ubiquitination and proteasome-mediated protein degradation [38]. These results demonstrate a probable relationship between oxidative damage and the production of protein aggregates that are hallmarks of Parkinson’s disease.

The outcomes of in vivo investigations of PD patients have been mixed. Ilic and colleagues have revealed that PD patients had elevated cerebrospinal fluid and blood amounts of malondialdehyde, along with the enhanced activity of glutathione reductase and Cu, Zn- superoxide-dismutase [37,38]. In PD patients’ leukocytes, serum, and cerebrospinal fluid, oxidative DNA damage has also been shown to be on the rise [39]. 

## 4. Ocular Manifestations in Parkinson’s Disease

Ocular symptoms are typically detected initially in the illness and might be classified as prodromal manifestations of PD; in fact, several researchers have discussed utilizing the existence of visual deficits as a viable diagnostic for prompt diagnosis of PD [40]. Progressive supranuclear palsy (PSP), corticobasal degeneration (CBD), and multiple system atrophy (MSA) all have ophthalmological clinical manifestations and ocular motor impairments. Their appearance is a key part of the process of diagnosing atypical Parkinsonism. 

### 4.1. Dopaminergic System and the Eye

Dopamine has a crucial role in a number of visual functions, including adaptation to light, oculomotor function, contrast sensitivity, color vision, visuospatial construction, and spatial working memory [41,42]. In Parkinson’s disease patients, a deficiency of dopamine may cause a variety of visual abnormalities, including diplopia. Patients with PD are also at higher risk for eyelid apraxia, blepharospasm, and dry eyes. 

Visual manifestations may be partially attributable to the well-documented existence of two kinds of dopaminergic neurons in the human retina: amacrine cells (inside the inner layer) and inner plexiform cells [43]. The earliest neurochemical findings of dopamine deficiency in the human retina corresponded with the loss of tyrosine hydroxylase immunoreactivity in the dopaminergic cells of the retina in a limited number of individuals with Parkinson’s disease [43]. Postmortem examination of untreated Parkinson’s disease patients revealed reduced retinal dopamine levels, whereas dopamine concentrations were normal in PD patients treated with a dopamine replenishing drug [44]. In addition, another study revealed the presence of misfolded [45] and phosphorylated alpha-synuclein [46] deposits in the retinas of PD patients, suggesting that, at minimum, a part of the visual impairment reported in these individuals may be attributable to the malfunction of the retinal network. Additional visual symptoms associated with Parkinson’s disease, including visual hallucinations and stereopsis anomalies, are produced by changes in cortical information perception [47,48]. Notably, MRI research revealed substantial changes in the pattern of the optic radiation connection in individuals with early PD [49].

Individuals with Parkinson’s disease may exhibit ocular movement symptoms, such as irregular saccades and smooth pursuit, in addition to visual processing issues caused by changes in the dopaminergic network at the retinal or cortical visual perceptual regions. Certain cortical and subcortical regions implicated in the progression of the disease could have a crucial role in creating part of the ocular motor impairment observed in PD [50,51].

In accordance with Braak’s neuropathology classification for PD, the engagement of the substantia nigra, which is believed to trigger the occurrence of motor clinical manifestations in PD patients, emerges slightly later in the progression of the disease (stage 3), while the initial engagement of the medulla and pontine tegmentum could give an explanation for certain initial non-motor symptoms [52]. This could clarify why non-motor clinical signs and symptoms appear prior to the motor phase of the illness. The occurrence of non-motor symptoms may be associated with more severe motor impairments and a worse quality of life [53]. Using radioligand binding studies and histology, dopamine and dopaminergic receptors in the retina have been revealed. Dopaminergic reduction in the retina of PD patients indicates the existence of neurodegenerative alterations in the retina. Reading difficulties, reduced visual acuity, color discrimination, motion perception, and contrast sensitivity are among the clinical deficits described by PD patients. Notably, levodopa may reverse the impairments in some of these clinical measurements, including contrast sensitivity.

### 4.2. Vision Disorders during Pd: Clinical Manifestations and Cellular and Molecular Mechanisms

In PD, modifications occur in all areas of the visual analyzer, including the eyeball and the exterior tissues. Therefore, a link between the alterations in the thickness of retinal layers was described. Also were noticed alterations in the frontal and occipital lobes in addition to the evolution of visual-spatial cognitive decline [54]. Dysfunction of the vision in PD typically manifests as diminished perceptual processing in the chromatic area, contrast adjustment, and/or visual acuity, along with a decreased perception of movement and a heightened incidence of hallucinations [54].

Hypokinesia may also affect the rate of blinking and the contract strength of muscle tissue (the orbicularis oculi muscle) in Parkinson’s disease patients. The above fibers encircle the meibomian gland channels and participate in the secretion of the meibomian gland. Additionally, the tear fluid circulation might shift, affecting the elimination of lipid secretion. Consequently, in these individuals, the meibomian glands situated in the eyelids might frequently have inflammatory responses, contributing to blepharitis and corneal abnormalities [55].

### 4.3. Color Perception

Deficits in color vision have been widely observed in untreated PD patients [56]. Motor impairments associated with the course of a disease may not accurately represent actual color limitations [57]. For example, some writers have argued that the color vision abnormalities found in PD patients are attributable in part to their general motor slowness [58]. Alternative assessments have also demonstrated color vision and contrast deficits in PD patients [59]. 

In particular, people with rapid eye movement sleep behavior disorder (an indication of alpha-synucleinopathies) who were not diagnosed with PD might have poor color vision [60]. In addition, color vision deficits in patients with rapid eye movement sleep behavior disorder are a significant predictor for illness progression [61]. According to research [62], PD patients with rapid eye movement sleep behavior disorder and color deficits might experience a more accelerated progression of the disease. Individuals with the LRRK2 gene mutation have also been investigated for color discrimination impairments in PD [63]. Surprisingly, these patients showed higher color deterioration compared to idiopathic PD patients [64].

There is a clear correlation between color vision abnormalities and illness duration [62], and levodopa medication might enhance color vision in certain PD patients [61]. Because of these results, some writers have suggested that problems with color vision could be an early sign of Parkinson’s disease [60]. 

### 4.4. Contrast Perception

Sensitivity to visual contrast is the capacity to differentiate a visual image from its backdrop. There are two forms of contrast sensitivity: spatial, for distinguishing static visual stimuli, and spatiotemporal, for tracking moving objects with the smooth following [65]. Patients with Parkinson’s disease exhibit diminished visual contrast sensitivity [66], which is most often observed in spatiotemporal contrast sensitivity, but other authors have found spatial contrast sensitivity to be significantly diminished [65]. Several investigations have demonstrated deficits over the full spatial-frequency spectrum [66], while others have demonstrated reduced contrast sensitivity exclusively in the intermediate-to-high frequency spectrum [67].

The deterioration of contrast sensitivity is associated with the course of the disease [68] and is partially recoverable with levodopa medication [69]. In instance, once PD patients with pronounced “on–off” variations are evaluated throughout their “on” state, their contrast sensitivity is comparable to healthy individuals of the same age [65].

Dopamine deficiency in the retina has been linked to color and contrast vision problems. Nevertheless, the pathogenesis of color vision loss in Parkinson’s disease is complicated and multifaceted [57]. Various investigations have shown that diminished contrast sensitivity in Parkinson’s disease is strongly linked with disease severity and cognitive decline, and that these manifestations could have prognostic or diagnostic relevance [68]. Also, an impeded color vision has been regarded as a presymptomatic indicator of neurodegeneration [69].

Significantly, levodopa medication might enhance contrast sensitivity and color vision in people with Parkinson’s disease. Additionally, specific absorption eyeglasses and enough ambient light might be helpful. Patients with diminished contrast sensitivity could have difficulty with scotopic vision and should thus be counseled against nighttime driving [70,71].

### 4.5. Saccades

Sometimes in the earliest stages of the illness, saccadic movement abnormalities are prevalent in PD patients; hence, several writers have proposed them as a feasible clinical diagnostic sign [72]. Electrooculographic measurements of individuals with Parkinson’s disease reveal abnormalities in saccadic and smooth pursuit eye movements [73]. Reflexive external (or eye-directed) saccades and voluntary internal (such as voluntary control, anticipatory, anti-saccadic, and memory-conducted saccades) are affected to differing extents in Parkinson’s disease.

Hypometric-reflexive and intentional saccades are the most common oculomotor disorders linked with Parkinson’s disease [73]. Patients with PD often approach their goal with a set of distinct, short saccades [73]. The hypometria observed in Parkinson’s disease patients is more prominent in memory-conducted voluntary saccades than in visually guided reflexive saccades [72]. Furthermore, patients with Parkinson’s disease have difficulty initiating memory-directed saccades, but this tendency is significantly preserved for visually led saccades [72].

In a study, it was suggested that dopaminergic failure may not be the primary cause of saccadic eye movement dysfunction in PD; instead, basal ganglia impairment may be the cause of the inability to quickly perform alternate voluntary gaze shifts [74].

Levodopa and dopamine agonists have contradictory effects on saccades; they decrease the delay of intentional saccades while simultaneously lengthening the latency of reflexive saccades. No important variations in saccadic amplitude have been recorded following levodopa medication for either visually or memory-led saccades [75].

### 4.6. Effortless Pursuit of the Eyes

Smooth pursuit eye movements (SPEM) are described as eye movements meant to track a moving target and maintain the picture in the fovea. Smooth pursuit eye movement anomalies could be observed in normal aging subjects, but they are a well-known observation in Parkinson’s disease [76]. Numerous cortical and subcortical regions, in addition to the particular brainstem and cerebellar areas, are implicated in their creation and regulation [77]. Initial and unmanaged PD patients demonstrated a decrease in pursuing gains [76]. A subpopulation of neurons in the substantia nigra pars reticularis (SNpr) exhibit a cessation of tonic firing during the beginning and persistence of smooth pursuit eye movements [78]. Its inappropriate activity might therefore, in theory, likewise clarify the prevalence of smooth pursuit eye movement abnormalities in PD patients.

Treatment with dopaminergic medications (bromocriptine or levodopa) has been linked to an enhancement in pursuit gain in individuals with Parkinson’s disease [77,78]. A second study found that dermal injections of Apomorphine had a significant benefit in 21 early untreated Parkinson’s disease patients [79]. Nevertheless, it must be emphasized that certain investigators did not find a significant enhancement in smooth pursuit eye movements following treatment with levodopa [80].

### 4.7. Square-Wave Jerks

Square-wave jerks (SWJ) are characterized as saccadic incursions that cause the eyes to depart from the fixation objective, accompanied by a correction saccade to the objective [81]. Despite being found in healthy subjects [82], square-wave jerks are frequently more pronounced in a number of neurological illnesses, including parkinsonian syndromes [81]. At the beginning of PD, square-wave jerks with an average amplitude of 2° were recorded [83]. This data contradicts the commonly held belief that square-wave jerks >1° occur more often in parkinsonian illnesses other than PD, including PSP and MSA [81]. Notably, higher amplitude and frequency of square-wave jerks have been recorded in PD patients following unilateral pallidotomy [84]. The main cause for problematic square-wave jerks is incompletely understood; abnormal square-wave jerks may be connected to the dysregulation of the fastigial area in the cerebellum, or they may be the consequence of higher activation in the frontal eye fields in Parkinson’s disease patients [83].

### 4.8. Convergence Inadequacy

Convergence inadequacy is a binocular-alignment disorder defined by the absence of eye convergence while focusing exclusively on close targets, leading to a distant close point of convergence and exotropia [85]. Various studies indicate that PD patients have a lower convergence amplitude compared to healthy subjects [86,87]. Convergence amplitude and near point of convergence are greater in PD patients who receive treatment compared to unmedicated patients, indicating that levodopa may improve this impairment [88].

Patients with symptoms of convergence insufficiency but a standard near the point of convergence were observed to have diminished convergence amplitudes [86,87]. These individuals demonstrate that asthenopia coupled with convergence insufficiency may develop in the absence of a near point of convergence that is sufficiently prolonged. In particular, these individuals emphasize the importance of measuring convergence amplitudes and not relying just on the near point of convergence when convergence inadequacy is suspected. Patients with lower convergence amplitudes and a normal near point of convergence often have adequate ocular movement, except for a difficulty to converge effectively. In addition, they are younger than individuals with complete convergence insufficiency syndrome, which suggests that these individuals would acquire convergence insufficiency with lower convergence amplitudes and a prolonged near the point of convergence later in their lives. 

Convergence deficiency coupled with presbyopia is commonly caused by accommodating convergence reduction. The induced base-out prism effect of bifocal lenses adds to presbyopic convergence insufficiency. PD patients, the majority of whom use bifocals, may have diminished accommodative capacity and be more sensitive to the negative effects of corrective lenses.

PD is linked with aberrant ocular movement, which results in impaired vision, diplopia, difficulty with close tasks, and limited gazing. Possible anomalies of eye movement include deficiencies in smooth pursuit, saccades, and vergence movements. In PD, movement planning and execution are hindered, and initiating pursuit is problematic. This may result in cogwheel motions. 75% of PD patients have impaired saccadic and smooth pursuit motions [89], significantly impacting focusing and reading. The optimization of dopaminergic drugs may enhance ocular motility [79]. Vergence eye movements function to shift the eyes in different orientations (whether towards, which would be converging, or separated, which is diverging), maintaining the stability of a picture on the retina as it travels towards or far from our eyes. To verify convergence, one might utilize the near point of convergence test. Inadequate convergence may hamper accommodation, the eye’s reaction to a close stimulus. When performing close activities, inadequate or sluggish accommodation may lead to visual discomfort, headaches, and diplopia. Twenty to thirty percent of PD patients suffer from diplopia due to abnormal convergence [90,91,92]. 

Referral to an ophthalmologist is recommended whenever the patient has difficulties in everyday living or when test findings are unsatisfactory, with a specific focus on convergence inadequacy. Patients with this condition may be corrected with prisms, which accommodate poor convergence, or with convergence exercises. Dopaminergic medications may help improve convergence impairment. In addition, physical and occupational specialists may utilize basic behavioral modifications to enhance visual performance. Examples of easy solutions are the use of monocular occlusion when reading, reading supports, and eating from high tables whenever there is a gaze constraint.

### 4.9. Diplopia

The incidence of diplopia in PD patients ranges from 10 to 47%, corresponding to a maximum of 19% in normal controls [11]. It is essential to distinguish between monocular diplopia (in one eye and often produced by intraocular disease), binocular diplopia (in both eyes caused by ocular misalignment), and selective double vision (a rare condition of visual hallucinations in Parkinson’s disease individuals). PD may cause binocular diplopia due to convergence deficiency or decompensated underlying strabismus. In latent strabismus, the eyes’ fusion process compensates for a relative deviation of the visual axis. Once this system is affected, the deviation manifests as diplopia. Disturbance could result from factors such as poor accommodation or weariness. In particular, diplopia could be detected as an “off” sign in individuals with reaction variations, suggesting an “off” phase [93].

Diplopia is more prevalent in people with pre-existing ocular misalignment, which appears to worsen as PD progresses. This could be due to a minor oculomotor dysfunction or a severe latent strabismus. Once the misalignment is detected, an assessment by an orthoptic ophthalmologist is recommended. Additionally, abnormal eye movements might affect depth perception (stereopsis). Stereopsis is the capacity to perceive the spatial relationships between things in three dimensions. To appropriately interpret things, a person essentially requires a healthy retina, adequate visual acuity, and properly aligned eyes. Research suggests a 42% incidence of stereopsis in PD [94,95].

### 4.10. Blepharospasm and Blepharitis

Blepharospasm and apraxia of eyelid movement are often seen in Parkinson’s disease patients [68]. Despite the possibility that extrapyramidal malfunction contributes to the higher prevalence of blepharospasm, ocular surface irritation could possibly play a role [69]. Before beginning expensive therapies including botulinum toxin treatment, PD patients with blepharospasm must have intensive treatment for ocular surface irritation.

As a consequence of dry eyes or blepharitis, patients could have a foreign body sensation, impaired vision, itching, or a burning sensation. Blepharitis symptoms include crusty eyelids, redness or inflammatory process of the eyelids, and scratchy or red eyes [96]. According to autonomic dysfunction, seborrheic blepharitis (similar to the more common seborrheic dermatitis) might develop, causing irritation and inflammation of the upper and lower eyelids. Ocular surface inflammation could indeed develop. This disorder may worsen the symptomatology of dry eyes, which are believed to be associated with an incidence of up to 60% in PD [86]. The impairment of the sebaceous Meibomian gland in the eyelids may result in a rise in dry eye and blepharitis manifestations [97].

### 4.11. Ocular Tremor

Gitchel et al. confirmed the occurrence of ocular oscillations in a significant number of PD patients [98]. During focusing exclusively on an object, the patients exhibited aberrant eye movements with a rhythmic sequence and a median-determined rate of 5.7 Hz, which is roughly inside the limit of PD tremor (4–8 Hz) [99]. Individuals diagnosed with essential tremors did not exhibit a comparable ocular tremor [98]. Several researchers have described these results as an oscillating vestibulo-ocular reflex generated by head tremors since these ocular oscillations were in the opposite direction from the pattern of head tremors [100]. Additional research might reveal how these results relate to disease development and if the dopaminergic medication affects these symptoms.

### 4.12. Reduced Blinking Rate

Reduced blinking rate is an additional factor leading to dry eyes. Normal blinking is considered to be approximately 20 to 30 blinks per minute, which may fluctuate while working, talking, or relaxing. The blinking rate might decrease as a result of medicine, weariness, disease, or aging. Dopamine regulates the motor regulation of blinking, which indicates why the blink rate can drop to as few as one to two times per minute in Parkinson’s disease [101]. Considerably fewer blinks can result in a less stable tear film due to evaporation, creating dry eyes. Consequently, this could ultimately result in excessive blinking, wet eyes, and blepharospasm [11].

### 4.13. Stereopsis

Stereopsis, often defined as “depth perception”, is the visual capacity to detect the three-dimensional nature of the environment. It has been established that the extrastriate cortex is primarily responsible for stereopsis [47]. Further precisely, current research suggests that defective stereopsis may be connected with non-dominant extrastriate cortical atrophy [47]. De novo drug-naive Parkinson’s disease patients might exhibit stereopsis deficits [95]. PD patients with defective stereopsis had lower motor control and higher motor assessments on the Universal Parkinson’s Disease Rating Scale (UPDRS) than PD patients without stereopsis deficits. Furthermore, depth perception deficits in PD patients may also be associated with diminished color perception [58].

The existence of stereopsis anomalies in PD has been linked to a faster rate of cognitive deterioration and has been identified as a predictive factor for dementia at 2 years [102]. These findings indicate a link between stereopsis deficits and the development of illness [58]. 

### 4.14. Visual Contrast Acuity

Visual acuity is the capacity of the eye to discern the most detailed characteristics of an element. Impaired visual acuity may be a leading cause of recurrent hallucinations in people with Parkinson’s disease [103]. The decline of amacrine cells and aggregation of alpha-synuclein in the retina have been investigated as potential causes of visual contrast acuity in patients with PD. In 2015, Lin et al. described the utilization of a novel iPad program to evaluate low contrast acuity in PD patients; it might serve as a complementary diagnostic instrument for individuals with PD and other neurological disorders [104].

### 4.15. Pupil Reactivity

Dopamine influences the activity of the smooth muscles of the iris, which control pupil size. In individuals with Parkinson’s disease, the characteristics of the pupil’s responsiveness to light are changed; tests on mice have demonstrated that exogenous dopamine produces pupil dilation, which is influenced by the dosage. This phenomenon is considered to be generated by the transformation of dopamine to norepinephrine, which dilates the pupil by activating the adrenoreceptors in the iris musculature [58]. Utilizing pupillometry, it was demonstrated that the proportion of constriction in the smooth muscles of the iris is decreased and that pupil constriction in reaction to a light stimulus is increased in Parkinson’s disease patients [58]. Surprisingly, these changes are more pronounced in people with Parkinson’s disease who are cognitively impaired than in those who are not [54]. Importantly, pupillary hyperreactivity to parasympathomimetic and sympathomimetic effects is a defining feature of PD, which may be utilized to detect signs of the preclinical stage of PD and evaluate therapy efficacy [54]. The noninvasive approach of pupillometry could be useful for establishing early detection of Parkinson’s disease.

Patients with PD were reported to have a larger pupil diameter and uneven pupil diameters following light adaptation [105]. Additionally, prolonged light reflexes with delays and constriction periods have been seen, whereas contraction amplitudes could be diminished, indicating a commitment dysfunction of the parasympathetic system in PD [103]. In PD, the iris muscle has the greatest capacity for contraction in comparison to healthy individuals, which shows that the muscle has developed adaptive sensitivity alterations [106].

### 4.16. Cornea in PD

The corneal area in PD also undergoes significant alterations. Patients with PD have a lower corneal thickness, presumably due to a reduced blinking rate and the appearance of dry eye syndrome [107]. Misra et al. revealed that the subbasal nerve plexus surface area in the cornea of PD patients was considerably lower than that of the controls [108]. The magnitude of the decrease in subbasal nerve fiber density in the cornea corresponds with the severity of cognitive decline [108,109]. It is probable that corneal neuropathy abnormalities precede motor performance deficits in Parkinson’s disease [110].

### 4.17. Retina in PD

Diverse visual deficits are caused by the retinal modifications identified in PD [111]. The production of cytotoxic aggregates of the neuronal protein alpha-synuclein exerts a major consequence in the development of Parkinson’s disease; the above aggregates are the primary constituent of Lewy bodies, a key diagnostic indicator of Parkinson’s disease. Alpha-synuclein accumulation in neurons is detected in the retinas of Parkinson’s disease patients, indicating that this molecule is turned into a toxin that causes neuronal damage, and may be a cause of visual malfunction [44]. Phosphorylated alpha-synuclein, which concurrently builds in the brain, was identified in retinal autopsy samples from PD patients [6,112]. Both dysfunctional phosphorylation and intracellular aggregation of alpha-synuclein are major elements and indicators in the etiology of Parkinson’s disease.

Individuals with Parkinson’s disease have a substantial bioelectric impairment (deficient impulse propagation across retinal neurons) of the visual pathway in the outer retinal layers [113]. In addition, the layers of nerve fibers and ganglion cells, and the inner and outer plexiform layers, are noticeably thinner in the retinas of PD patients than in those of healthy individuals [114]. As PD progresses, the inner retinal layers grow thinner. Likewise, the ganglion cell layer varies [115]. Scotomas were discovered in PD patients even though the retinal thickness had not diminished [114]. Individuals who have PD have an abnormal fovea: the upper-lower inclination is flattened, and the nasal-temporal angle of inclination is narrower [116]. Patients with Parkinson’s disease have an irregular foveal retinal thickness between the eyes [117]. Optic degeneration is also seen in PD patients [113].

### 4.18. RNFL and OCT Changes

Optical coherence tomography (OCT) allows for the non-invasive assessment of direct structural proof of retinal dysfunction in PD [60,61]. Later application of this procedure revealed a reduction in the inner retinal nerve fiber layer (RNFL) [118]. The narrowing of the retina seems to be more evident contralaterally to the side with motor symptoms and on the same side as the afflicted substantia nigra [119]. Moreover, a few investigations [120,121] did not find RNFL reduction in PD patients, but one research study [120] demonstrated that the outer nuclear and photoreceptor layers were thinner than in healthy subjects. Regarding variances in sectoral distribution, it is evident that people with PD have retinal thinning, largely in the inner retina. A detailed review of device characteristics, segmentation methods, and dependability, in addition to disease stage, could indicate why certain studies do not reveal substantial changes in pRNFL thickness [121,122]. Modifications in macular thickness may precede visible RNFL modifications of the ganglion cell body, dendritic field, and synaptic connections, which are the principal sites of cell destruction. Intriguingly, several investigations indicate changes in functional parameters in the absence of anatomical thinning, indicating that functional alterations could predate the detection of pRNFL thickness reduction [114].

In particular, the length and complexity of the disorder correspond with the depletion of the ganglion cell layer in the inner retina [123,124]. In PD, the thickness and density of the macular retina, such as the inner and outer plexiform layers, are diminished [123,124]. The amount of macular loss in Parkinson’s disease patients might correspond with the progression of the disease and clinical severity, as determined by the Hoehn and Yahr scale and the Unified Parkinson’s Disease Rating System, Part III (UPDRS III) [123]. Consequently, macular assessments using OCT may serve as an indicator for the course of Parkinson’s disease [124]. Previous analysis indicates that PD patients have a thicker choroid than healthy control subjects [123,124].

Researchers suggest that retinal thinning in Parkinson’s disease indicates a main reduction of dopaminergic amacrine cells and a drop in dopamine neurotransmitter levels in the retina, which probably mirrors comparable modifications in the CNS. This idea has functional ramifications, along with a link between retinal alterations and problems in visual function, motor function, and cognitive function. A meta-analysis revealed significant inner retinal layer thinning in PD. However, there were only a few correlations between retinal thickness and severity of symptoms, which could be attributed to differences in underlying data and methods across studies [115].

## 5. Ophthalmologic Diseases in Parkinson’s Disease

### 5.1. Dry-Eye Syndrome in Parkinson’s Disease

Dry-eye syndrome is common in Parkinson’s disease patients: tear secretion is reduced, and the lacrimal coating of the cornea is altered [125]. One cause would be decreased blinking rate, and the other cause would be the degeneration of the lacrimal, meibomian, and other glands. There was a statistically significant association seen between the degree of dry-eye symptoms and the progression of PD [126]. In individuals with Parkinson’s disease, enhanced lacrimation is frequently found simultaneously with dry eyes. Variations in eyelid movements might be the cause.

If dry eyes are caused by a lower blink rate or blepharitis symptoms, a referral to an ophthalmologist is recommended [127,128]. Artificial tears and eyelid cleanliness are conventional therapies for this condition however, dopaminergic medication dosage changes might be helpful [74,129]. In cases of blepharospasm, subcutaneous injections of botulinum toxin could be beneficial [130].

### 5.2. Parkinson’s Disease and Glaucoma

Patients with Parkinson’s disease have a 30% increased risk of developing glaucoma. Patients with glaucoma had a lower concentration of catecholamines, including dopamine, in aqueous humor and lacrimal fluid than normal subjects, indicating that glaucoma induces malfunction of the dopaminergic circuitry of the eye [54,131]. In addition to PD, glaucoma has a complex pathogenesis. It is possible that the evolution of neurodegeneration in Parkinson’s disease and the loss of ganglionic neurons in glaucoma involve similar pathogenetic processes, including oxidative stress and the engagement of microglia in the central nervous system [131].

### 5.3. Parkinson’s Disease and Cataract

Patients with PD are 1.48 times more likely to develop cataracts than the normal population [132]. In the lens of people with PD and cataract, glyceraldehyde-3-phosphate dehydrogenase function, which is implicated in glycolysis and apoptosis activation, is decreased compared to patients who have cataracts without having PD [132]. In particular, the lenses of PD patients collected following cataract surgery had a greater concentration of alpha-synuclein in comparison with those of cataract patients not having PD [133]. The increase of alpha-synuclein and its aggregates forming cytotoxic compounds in neurons of Parkinson’s disease patients and in the crystalline lens of cataract patients suggests that both diseases’ pathogenesis utilizes a commune pathway.

## 6. PD Visual Impairments and Quality of Life

Numerous PD patients report having visual problems that often limit their ability to do everyday tasks. Reading is comprised of serial fixations in addition to forward and backward saccades, according to the theory of eye movements. Because PD saccades are slow and seem to have a longer delay in PD patients who have trouble thinking, these things might affect the ability to read.

Due to a higher frequency of regressive saccades and longer average fixation lengths, PD patients read considerably fewer words per minute than controls. Slower reading rates in PD could be mostly due to cognitive impairment as opposed to motor dysfunction. Reading is a multifaceted activity requiring the integration of several cognitive areas. Regarding semantic and phonological processing, the cortical reading network is comprised of frontal and temporal areas [134]. In the initial stages of Parkinson’s disease, dopaminergic frontostriatal impairment, leading to degradation of executive functions, might be predicted [135], while visuospatial impairments might suggest cognitive decline leading to PD moderate cognitive problems or PD dementia [136]. The extended mean length of fixation in PD can indicate difficulties in processing visual data and planning the subsequent saccade. A greater number of regressive saccades may be a compensatory mechanism for integrating obtained data with information missed during the first reading [137], whereas the increasing variation of regression amplitudes with illness duration may be a substitute for cognitive impairment with visuospatial disorder. Patients with early Alzheimer’s disease have a similar reading style, confirming the theory of a cognitive origin of reading problems in Parkinson’s disease [138].

Due to their desire to visually compensate for their impairments through spontaneous, internally produced motions, people with PD are especially susceptible to visual problems. For instance, visual signals, including stripes on the floor, may be used to prevent gait freezing [139]. Consequently, an inability to effectively notice these visual signals could have an instant effect on everyday functioning. In fact, visual impairments paired with postural instability and gait impairment might raise the likelihood of falling and sustaining trauma, including hip fractures [140]. Lastly, visual problems impede everyday activities and can therefore result in social isolation [141]. Therefore, visual abnormalities negatively affect the quality of life of PD patients [1,142].

Using the visual deficits test in conjunction with particular tests to evaluate color discrimination and contrast sensitivity, along with other non-motor sectors including sleep irregularities, hyposmia, dysautonomia, and depression, indicated a remarkable selectivity in distinguishing early PD patients from healthy participants [40]. Several of these visual anomalies might appear not just during the initial stages of the illness, but also prior to its beginning.

The existence of visual impairments at the beginning of the disease process might serve to anticipate the disease process: color vision deficits are related to an elevated risk of dementia in Parkinson’s disease (PD) patients [143], while stereopsis defects are associated with a faster cognitive impairment [102]. Consequently, the existence of visual anomalies not only could help as an initial indicator of Parkinson’s disease but also as a predictor of disease complications and prognosis.

Visual deficiencies in Parkinson’s disease may impact total motor performance [40], resulting in postural instability, an increased frequency of falls, and a deteriorating quality of life; lower contrast sensitivity has been linked to a higher risk of frozen gait [9]. Individuals with the tremor-predominant phenotype, on the other hand, had fewer color vision impairments and a milder outcome [62]. The processes causing these phenotypic discrepancies are uncertain. The presence of concurrent brain pathology or variable brain location of α -synuclein deposits in these individuals may explain the phenotypic disparities [62]. 

As the condition advances, visual abnormalities develop. Deficiencies in color vision, contrast sensitivity, and stereopsis are associated with the progression of the disease [84]. Accurate information on the effect of dopaminergic treatment on visual clinical manifestations is also provided; levodopa enhances color vision and contrast sensitivity in PD patients [144] and may affect saccades [75]. These results reinforce the idea that visual problems are associated with the well-documented decline of dopaminergic neurons inside the retina, suggesting that at minimum certain manifestations may be addressed by an established modification in the flow of visual input at the retinal surface.

Individuals with rapid eye movement sleep behavior disorder who had not yet been diagnosed with Parkinson’s disease had altered color vision [145]. Furthermore, color vision deficits in rapid eye movement sleep behavior disorder patients have been recognized as a risk factor for illness progression [62]. Consequently, color vision deficits could be an initial diagnostic indicator of Parkinson’s disease [146,147].

## 7. Management and Therapy

Patients with PD often report ocular irritation and the perception that “something is off” with their vision. In consequence, both the physician and the patient could experience dissatisfaction, as a definitive diagnosis is often challenging. By detecting the typical, yet sometimes misdiagnosed, ocular symptoms of early PD, clinicians may concentrate on the most effective therapy and reassure patients. Sometimes, basic treatments may achieve amazing outcomes. The doctors should eliminate pharmaceuticals that interfere with tear production and accommodation. Blepharitis and other diseases of the ocular surface must be addressed. The use of artificial tears to adequately lubricate the cornea should be recommended [148,149]. Patients with tremors may benefit from reading with a book holder. As contrast sensitivity and color vision could be impaired, patients must have adequate ambient light when reading. Detecting and treating convergence deficiency and reduced convergence amplitudes. A base-in prism could be required for reading glasses. If appropriate single vision cannot be achieved even with prismatic correction, monocular occlusion during reading might offer a solution. 

**Figure 1:**
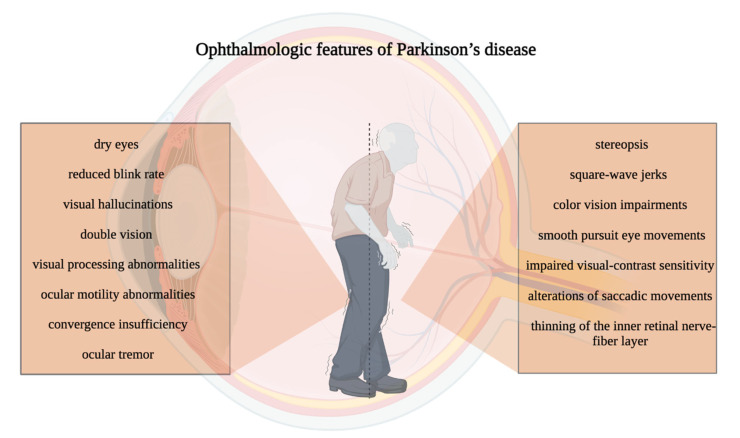
Ophthalmologic features of Parkinson’s disease.

Patients with asthenopia or diplopia ought to undergo a thorough refraction and may benefit from avoiding bifocals or progressive lenses. It may be beneficial to have separate glasses for distance, reading, and computer usage. The spherical equivalent may be preferred to extensive astigmatic correction when prescription eyeglasses are needed for individuals with tremor, dyskinesias, or a history of falls. Patients should be advised to use a finger to move their eyes around the page when their saccadic velocity is diminished. In order to identify modest central hemianopia abnormalities, formal visual fields must be collected from patients who have had a pallidotomy and who claim reading difficulties. Blepharospasm and apraxia of eyelid movement must be addressed with botulinum toxin and/or operations if they do not ameliorate following ocular surface irritation therapy. In Parkinson’s disease, visual hallucinations can occur suddenly and need not necessarily result in therapy modifications. By recognizing the precise causes of patients’ concerns and providing therapy, possibly frustrating physician–patient interactions can be avoided in support of a pleasant therapeutic team. Ophthalmologic features of Parkinson’s disease can be found in Figure 1.

## 8. Conclusions

Transitions happened in all components of the eye and the eye’s auxiliary system throughout PD. The retina, which shares a developmental genesis with the central nervous system, is susceptible to neurodegenerative disorders. These disorders may occur in the tissues of the anterior chamber of the eye, which regulate intraocular pressure and accommodation and are essential for the diameter of the pupil. Substantial modifications appear on the surface of the eye: dry eyes and blepharitis, which often induce corneal thinning, are present. Additionally, the cornea’s subbasal nerve fiber quantity diminishes. 

Contemporary ophthalmological diagnostic techniques enable noninvasive detection of the abnormalities and may be effective in establishing an early diagnosis of Parkinson’s disease. In addition to ophthalmological evaluation, it is also feasible to explore the lacrimal fluid for indicators of PD. The data provided in this article on the correlation between neurodegenerative activity in the brain and the eye enables the visualization of the eye as a “window” that creates the opportunity to recognize initial symptoms of Parkinson’s disease and to accurately predict novel pathophysiologic mechanisms that underlie the progression of neurodegeneration in the eye on its own.

In summary, our analysis demonstrates that visual and oculomotor abnormalities are prevalent in PD, even in its earliest stages. Several of these signs might worsen as the illness progresses, and levodopa medication might affect the severity of a great number of symptoms. Certain visual disorders may represent initial symptoms of Parkinson’s disease or even early clinical indicators. For instance, multiple studies indicate a link between modifications in the retina and pathological brain alterations in Parkinson’s disease, suggesting the retina as a possible biomarker for the diagnosis of PD considering the reduction of dopamine neurons in the retinal cells of Parkinson’s disease patients. Neuropathological, morphological, and electrical abnormalities in the PD retina, as well as phosphorylated Alpha-Syn aggregation in the PD retina, provide evidence that the retina may serve as a biomarker for Parkinson’s disease. The evaluation of the retina in PD patients can be easily performed through optical coherence tomography, which is an investigation that usually discovers the abnormalities of the retina that are linked to the PD. Nevertheless, more imaging investigations are required to validate these observations. Consequently, recognition of these indicators may provide a faster medical assessment of PD and may also anticipate the disease prognosis, resulting in a higher quality of life for patients.

## Data Availability

Not applicable.
